# Editorial: Biofabrication and Biopolymeric Materials Innovation for Musculoskeletal Tissue Regeneration

**DOI:** 10.3389/fbioe.2022.909577

**Published:** 2022-05-24

**Authors:** Megan E. Cooke, Derek H. Rosenzweig, Chaozong Liu, Farnaz Ghorbani

**Affiliations:** ^1^ BioFrontiers Institute, University of Colorado at Boulder, Boulder, CO, United States; ^2^ Department of Surgery, The Research Institute of McGill University Health Centre, McGill University, Montreal, QC, Canada; ^3^ Institute of Orthopaedic and Musculoskeletal Science, Royal National Orthopaedic Hospital, University College London, London, United Kingdom; ^4^ Institute of Biomaterials, University of Erlangen-Nuremberg, Erlangen, Germany

**Keywords:** bioprinting, biofabrication, biomaterials, biopolymers, tissue engineering, musculoskeletal

The human musculoskeletal system provides form, support, stability, and movement to the body. It is made up of the bones of the skeleton, muscles, cartilage, tendons, ligaments, joints, and other connective tissues. The primary functions of the musculoskeletal system include supporting the body, allowing motion, and protecting vital organs. The skeletal portion of the system serves as the main storage system for calcium and phosphorus and contains critical components of the hematopoietic system (Li and Niu, 2020). Musculoskeletal Disorders (MSDs) include injuries and diseases that primarily affect the movement of the human body. They are characterized by pain and limitations in mobility, dexterity, and overall level of functioning, reducing patients’ ability to work and maintain a good quality of life. A recent analysis of Global Burden of Disease data showed that approximately 1.71 billion people globally have musculoskeletal conditions (Woolf and Pfleger, 2003). MSDs such as osteoarthritis, rheumatoid arthritis, psoriatic arthritis, gout, and ankylosing spondylitis affect joints (McInnes and Schett, 2011; Loeser et al., 2012; Litwic et al., 2013); osteoporosis, osteopenia and associated fragility fractures, as well as traumatic fractures, affect bones (Florencio-Silva et al., 2015); sarcopenia affects muscles, and back and neck pain affect the spine of the human body.

Tissue engineering is a concept whereby cells are taken from a patient, their number is then expanded before being seeded on a biomaterial scaffold. The appropriate stimuli (chemical, biological, mechanical and electrical) are applied, and new tissue is formed over time. This new tissue is then implanted to help restore function for the patient (Liu et al., 2007). To achieve the repair and regeneration of musculoskeletal tissues is still a challenge that requires the combined effort of biomaterials scientists, tissue biologists, and engineers. Material selection is critical to ensuring that cell-seeded tissue constructs have appropriate mechanical and biological environments.

Biopolymers are natural materials derived from plants and animals including polysaccharides such as alginate, chitosan, hyaluronic acid, and polypeptides such as gelatin, silk fibroin and elastin. Many biopolymers have properties such as cell adhesion and degradability and form highly swollen networks that provide physiologically relevant environments for cell culture (Muir and Burdick, 2021). Biopolymers can also be chemically functionalised to bring about control over their cell-binding and cross-linking capabilities (Muir and Burdick, 2021). In TE of soft MSK tissues, such as cartilage, ligaments and intervertebral discs biopolymer hydrogels have been extensively used as they provide a highly hydrated 3D matrix for these largely avascular tissues (Kesti et al., 2015). Bone is the hard tissue of the musculoskeletal system and is a commonly investigated tissue for regeneration. Tissue engineering approaches are usually combinatorial between hard and soft materials to produce composite scaffolds for cell attachment with load-bearing capacity, and often osteogenic or osseointegrative cues.

A common material for spinal cages is PEEK (polyether-ether-ketone) but the lack of osseointegration is a concern. Li et al. produced a nanohydroxyapatite/polyamide 66 composite which showed better osseointegration, as demonstrated by push-out tests in a rabbit femoral condyle model. Higher forces required to push out the new composite indicate better bone-implant integration. Ghorbani et al. developed polydopamine microspheres with unique, pomegranate-like morphology. Using self-oxidative polymerization of dopamine hydrochloride and precise pH control, porous microspheres were produced from agglomerated nanospheres. Molecular calculations then demonstrated that this material could interact with BMP2, Decorin, and Matrilin-1, enabling the formation of a protein layer that could be utilised in bone and cartilage tissue engineering. Polyhydroxyalkanoates are biopolyesters produced by microbes under unbalanced culture conditions. Their biocompatibility and biodegradability *in vivo* make them attractive materials for tissue engineering. Li et al., review their synthesis, properties and applications in bone tissue engineering, including manufacturing through 3D printing.

Musculoskeletal tissues have mechanical functions, so introducing mechanical loading during culture is used to increase extracellular matrix production in tissue-engineered constructs. Hart et al. present a perspective on the challenges of regenerating these tissues that natively reside in unique biomechanical environments. A key takeaway is that anabolic cues are present in the growth and maturation of these tissues, so replicating the *in vivo* loading environment during the maturation of a tissue construct *in vitro* is likely to be beneficial. Loading regimes in cartilage TE were reviewed by Sardroud et al., in the context of undesirable fibrocartilage formation. Collagen type II is found in native cartilage, but there is usually a combination of collagen types I and II in tissue-engineered cartilage. This forms fibrocartilage, a mechanically inferior form of cartilage. Of particular interest is the literature on the mechanotransduction pathways that lead to this fibrocartilage formation. Ge et al. studied the effects of mechanical compression in driving chondrogenesis in agarose hydrogels. They found that dynamic mechanical loading of synovial MSC-agarose constructs on day 1 of culture resulted in unwanted markers, but when loaded on day 21 expression of chondrocyte-specific markers was increased and hypertrophy markers were decreased. The host body response of tissue-engineered cartilage was reviewed by Wei et al. Most implanted materials will activate a response from the innate and adaptive immune systems. This guides a remodelling process that when understood may be beneficial to promoting cartilage regeneration and better integration of implanted tissue constructs. The authors consider synthetic and natural biomaterials, including a strong rationale for the use of a decellularized extracellular matrix to remove immune components that would otherwise cause a negative implantation response. Biopolymers for tissue engineering of other connective tissues were also investigated in this Research Topic. Li et al. reviewed advances in materials for intervertebral disk regeneration. These include polymers such as chitosan and collagen that, in combination with growth factors and cell transplantation show promise for endogenous regeneration. Kang et al. investigated polydopamine as a coating to immobilise BMP-2 on PET scaffolds for ligament tissue engineering. Modified grafts significantly improved bone integration in a rabbit model compared to PET alone.

Bioprinting, defined as “*the use of computer-aided transfer processes for patterning and assembling living and non-living materials with a prescribed 2D or 3D organization in order to produce bioengineered structures serving in regenerative medicine, pharmacokinetic and basic cell biology studies”* (Guillemot et al., 2010) is a rapidly growing field of tissue engineering. Bioprinting gives exceptional control over the spatial deposition of materials, cells, and other factors to enable the production of both implantable materials as well as *in vitro* tissue models for personalized medicine and drug screening applications (Cooke and Rosenzweig, 2021). Importantly the use of an additive manufacturing process enables the production of patient-specific geometries without the use of traditional subtractive manufacturing processes. Biomaterial inks, used as materials in bioprinting, must meet specific rheological parameters to ensure extrudability as well as rapid shape recovery to produce high fidelity constructs. With the addition of cells and other biological materials, the term bioink is used to describe these materials (Groll et al., 2018). Through bioink and application developments, bioprinting has been applied to a range of tissue-like constructs including soft, hard, and interfacing musculoskeletal tissues (Moxon et al., 2017; Alcala-Orozco et al., 2020).

Several articles in this issue consider the use of bioprinting for connective musculoskeletal tissues. Aerosol jet printing was employed by Gibney and Ferraris to produce droplets of collagen types I and II with size ranges less than 5 um. These were then extruded through a nozzle to print dense scaffolds, 576 layers high that resulted in aligned scaffolds post-neutralization. These dense scaffolds strongly replicate the native dense ECM of connective tissues. In another connective tissue study by Li et al., a PCL template scaffold was printed before being injected with a meniscal extracellular matrix. The addition of kartogenin-loaded microspheres was shown to increase chondrogenesis of synovium-derived MSCs *in vitro*. Increased secretion of total collagen and aggrecan show that this is a promising scaffold for meniscal tissue engineering. Lan et al. produced a bioink of meniscal fibrochondrocytes in a TEMPO-oxidized alginate. Following rheological characterization to optimize the formulation, they were printed into discs and cultured in low oxygen conditions to mimic the avascular meniscus environment. Histologically and biochemical analyses showed that compared to collagen type I control constructs, the TEMPO-alginate scaffolds had significantly higher COL2A1 expression and more meniscal-like phenotypes. There was clear increased production of aggrecan histologically in the TEMPO scaffolds. A review of recent trends in biofabrication for skeletal muscle disease modelling investigated the other aspect of bioprinting technology, to investigate diseases in more physiologically relevant culture systems than common 2D monolayer cultures. Cho and Jang discuss studies that encourage uniaxial cell alignment, as is observed in skeletal muscle, and how different biofabricated *in vitro* models have been used to replicate muscular dystrophies and inflammatory diseases.

There are still many challenges facing clinical translation of bioprinted tissues/organs such as bone, cartilage, muscle, or ligaments/tendons. Two main challenges include mechanical integrity and vascularization of generated tissues. Much progress is being made using gel-in-gel printing strategies to incorporate vasculature in bioprinted tissues. However, mechanical integrity is often overlooked. Current gold standard treatments of autografts and allografts (e.g., ligament and bone) consider mechanics and nutrient supply to the tissue. Prostheses for total joint replacement do not require vascularization but possess appropriate biomechanical properties. Therefore, the future of bioprinted tissues for MSK repair and regeneration will depend on the advancement of tissue maturation with increased mechanical strength, and improved methods for vascularization for nutrient supply upon implantation. Much progress has been made in scaffold design and compartmentalization but fully functional human anatomic biofabricated organs are still perhaps many years away from being realized. Perhaps the most practical current use of bioprinted human MSK-like constructs lies in screening novel therapeutics and better understanding the mechanisms of disease. Nonetheless, the emergence of bioprinting is an inspiring and exciting advance in the field of tissue engineering.

## References

[B1] Alcala-Orozco C. R. Mutreja I. Cui X. Kumar D. Hooper G. J. Lim K. S. (2020). Design and Characterisation of Multi-Functional Strontium-Gelatin Nanocomposite Bioinks with Improved Print Fidelity and Osteogenic Capacity. Bioprinting 18, e00073. 10.1016/j.bprint.2019.e00073

[B2] Cooke M. E. Rosenzweig D. H. (2021). The Rheology of Direct and Suspended Extrusion Bioprinting. Apl. Bioeng. 5, 011502. 10.1063/5.0031475 33564740 PMC7864677

[B3] Florencio-Silva R. Sasso G. R. d. S. Sasso-Cerri E. Simões M. J. Cerri P. S. (2015). Biology of Bone Tissue: Structure, Function, and Factors that Influence Bone Cells. BioMed Res. Int. 2015, 1–17. 10.1155/2015/421746 PMC4515490 26247020

[B4] Groll J. Burdick J. A. Cho D.-W. Derby B. Gelinsky M. Heilshorn S. C. (2018). A Definition of Bioinks and Their Distinction from Biomaterial Inks. Biofabrication 11, 013001. 10.1088/1758-5090/aaec52 30468151

[B5] Guillemot F. Mironov V. Nakamura M. (2010). Bioprinting Is Coming of Age: Report from the International Conference on Bioprinting and Biofabrication in Bordeaux (3B'09). Biofabrication 2, 010201. 10.1088/1758-5082/2/1/010201 20811115

[B6] Kesti M. Eberhardt C. Pagliccia G. Kenkel D. Grande D. Boss A. (2015). Bioprinting Complex Cartilaginous Structures with Clinically Compliant Biomaterials. Adv. Funct. Mat. 25, 7406–7417. 10.1002/adfm.201503423

[B7] Li G. Niu W. (2020). “Challenges toward Musculoskeletal Injuries and Diseases,” in Nanoeng. Musculoskelet. Regen. (Academic Press), 1–41. 10.1016/b978-0-12-820262-3.00001-3

[B8] Litwic A. Edwards M. H. Dennison E. M. Cooper C. (2013). Epidemiology and Burden of Osteoarthritis. Br. Med. Bull. 105, 185–199. 10.1093/bmb/lds038.Epidemiology 23337796 PMC3690438

[B9] Liu C. Xia Z. Czernuszka J. T. (2007). Design and Development of Three-Dimensional Scaffolds for Tissue Engineering. Chem. Eng. Res. Des. 85, 1051–1064. 10.1205/cherd06196

[B10] Loeser R. F. Goldring S. R. Scanzello C. R. Goldring M. B. (2012). Osteoarthritis: A Disease of the Joint as an Organ. Arthritis & Rheumatism 64, 1697–1707. 10.1002/art.34453 22392533 PMC3366018

[B11] McInnes I. B. Schett G. (2011). The Pathogenesis of Rheumatoid Arthritis. N. Engl. J. Med. 365, 2205–2219. 10.1056/NEJMRA1004965 22150039

[B12] Moxon S. R. Cooke M. E. Cox S. C. Snow M. Jeys L. Jones S. W. (2017). Suspended Manufacture of Biological Structures. Adv. Mat. 29, 1605594–1605596. 10.1002/adma.201605594 28145596

[B13] Muir V. G. Burdick J. A. (2021). Chemically Modified Biopolymers for the Formation of Biomedical Hydrogels. Chem. Rev. 121, 10908–10949. 10.1021/acs.chemrev.0c00923 33356174 PMC8943712

[B14] Woolf A. D. Pfleger B. (2003). Burden of Major Musculoskeletal Conditions. Bull. World Health Organ. 81, 646–656. 14710506 PMC2572542

